# Clinical outcome and radiographic change of ipsilateral scapular neck and clavicular shaft fracture: comparison of operation and conservative treatment

**DOI:** 10.1186/s13018-014-0141-0

**Published:** 2015-01-28

**Authors:** Tsung-Li Lin, Yu-Fen Li, Chin-Jung Hsu, Chih-Hung Hung, Chi-Chang Lin, Yi-Chin Fong, Horng-Chaung Hsu, Chun-Hao Tsai

**Affiliations:** Department of Orthopedic Surgery, China Medical University Hospital, #2 Yue-Der Road, Taichung, 40447 Taiwan; Graduate Institute of Biostatistics, China Medical University, Taichung, Taiwan; School of Chinese Medicine, China Medical University, China Medical University Hospital, Taichung, Taiwan; Graduate Institute of Clinical Medical Science, School of Medicine, China Medical University, Taichung, Taiwan

**Keywords:** Scapular fracture, Clavicle fracture, Floating shoulder, Glenopolar angle

## Abstract

**Objective:**

The purpose of this study is to compare glenopolar angle (GPA) and the functional outcomes of fixation of both the clavicle and the scapular neck, fixation of the clavicle alone, and conservative treatment for floating-shoulder injuries.

**Methods:**

A prospective stratified randomized study was performed in 39 adult patients who suffered floating-shoulder injuries and underwent fixation of both the clavicle and the scapular neck (group A), or fixation of the clavicle alone (group B), or conservative treatment (group C) between January 2005 and September 2011. The GPA, Disabilities of the Arm, Shoulder and Hand (DASH) score, and Constant-Murley Shoulder Outcome (Constant) score were compared between the three groups.

**Results:**

All 39 patients were followed up for more than 2 years. GPA after bony consolidation was significantly better in group A than in groups B and C (*p* = 0.015). Functional outcomes measured by DASH and Constant scores were significantly better in group A at final follow-up (*p* = 0.008 and 0.002, respectively). Both DASH and Constant scores were highly correlated with GPA after consolidation (*p* < 0.001, respectively). The receiver operating characteristic (ROC) analysis showed that of the two randomly selected DASH scores, the smaller DASH score would have a larger GPA than the larger DASH score. Similarly, the larger Constant score would have a larger GPA than the smaller Constant score.

**Conclusions:**

Fixation of both the clavicle and the scapular neck may correct GPA and improve functional outcomes for the treatment of floating-shoulder injuries. GPA after fracture consolidation is a useful prognostic indicator of a satisfactory clinical outcome as defined by either DASH score or Constant score.

## Background

Double disruption of the superior shoulder suspensory complex (SSSC) creates anatomic instability [1]. Floating-shoulder injuries, or ipsilateral fractures of the clavicle and the scapular neck, are double disruptions of the SSSC that render the entire shoulder girdle unstable [2,3]. Good results have previously been reported for both conservative treatment [3-5] and operative treatment of the clavicle alone [2,6-8] or of both the clavicle and the scapular neck [9]. There have been no randomized trials because the condition is rare; the criterion for surgery is based on radiologic evidence; and functional measurements are still under discussion.

The purpose of this prospective stratified randomized study was to analyze the clinical and radiographic outcomes of floating-shoulder injuries by comparing the outcomes of operative and conservative treatment. Our null hypothesis was that there were no differences between the fixation of both the clavicle and the scapular neck, fixation of the clavicle alone, and conservative treatment.

## Materials and methods

This study was approved by our institutional review board. Between January 2005 and September 2011, 39 consecutive adult patients were identified with floating-shoulder injuries. Inclusion criteria were age between 20 and 70 years and displaced ipsilateral clavicular shaft and scapular neck fractures. The definition of displacement of clavicular facture was 1) displacement greater than 10 mm or 2) shortening greater than 20 mm [10]. Displacement of scapular neck fracture was defined as 1) displacement greater than 20 mm, 2) angulation greater than 40°, or 3) the combination of angulation greater than 30° and displacement greater than 15 mm [11]. Exclusion criteria were 1) open fracture, 2) associated intra-articular glenoid fracture, 3) concomitant scapular process fractures, 4) proximal humeral fracture, 5) ipsilateral fracture of upper extremity, 6) associated neurovascular injury, and 7) history of dislocation or fractures or surgery around the affected shoulder.

The diagnosis of floating-shoulder injuries was made based on plain radiographs and computer tomography. The glenopolar angle (GPA) is a measurement of the rotational malalignment of the glenoid about an anteroposterior (AP) axis perpendicular to the scapular plane [12]. The GPA is the angle formed by the line connecting the most cranial and most caudal points of the glenoid cavity and the line connecting the most cranial point of the glenoid cavity with the most caudal point of the scapular body. Measurements of GPA were made at presentation from true AP views of the affected scapula and recorded at the time of initial diagnosis by three physicians (TLL, CCL, HCH).

**Figure 1:**
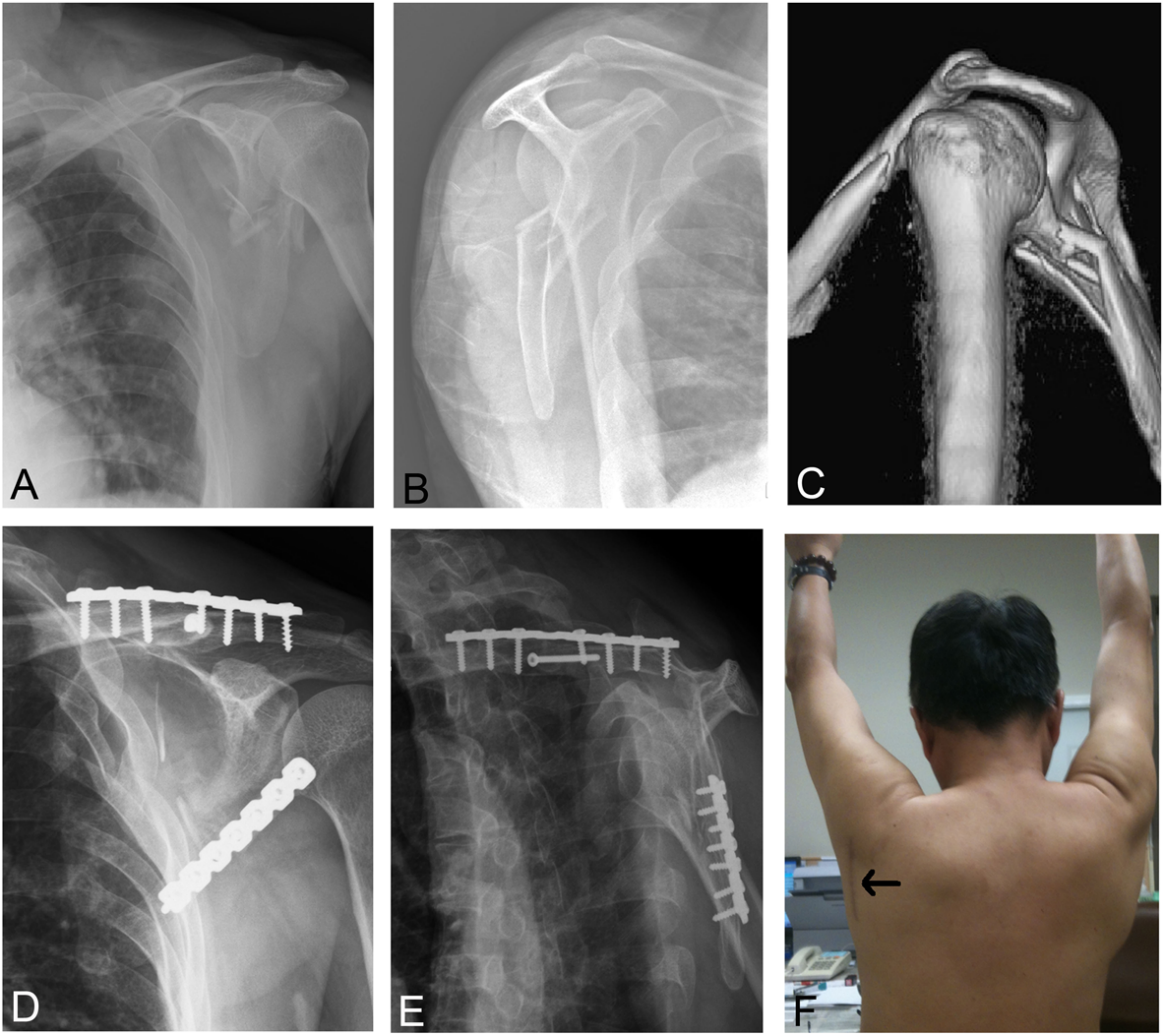
**Course of double fixation.** Anteroposterior **(A)** and lateral **(B)** radiographs of the patient with ipsilateral fracture of the clavicle and the scapula neck before the treatment. The glenopolar angle was 3°; three-dimensional computerized tomography image showed the scapular neck fracture angulation was up to 43° **(C)**; post-operative follow-up radiographs revealed the glenopolar angle was 36° **(D,** **E)**; the post-operative photograph showed the operated shoulder **(F)** and a straight incision over the glenoid neck to access minimal dissection between the teres minor and the infraspinatus (arrow).

Patients were divided into three groups under stratified randomized design: fixation of the clavicle and the scapular neck (group A), fixation of the clavicle alone (group B), and conservative treatment using a sling for 4 weeks (group C). In those patients who had been treated surgically, primary stabilization of the clavicle or the scapular neck was performed using either 3.5-mm dynamic compression plate or 3.5-mm AO reconstruction plate. Surgery was performed with the patients in the lateral decubitus position with the affected arm free for easy manipulation, and the clavicular fracture was repaired by attaching the plate to the superior aspect of the clavicle. Plate repair of scapular neck fractures was performed using a less invasive approach after osteosynthesis of the clavicular fracture in group A. A less invasive approach was chosen for fixation of scapular fractures which is less soft tissue dissection, muscle destruction, and blood loss than in the traditional Judet approach [13]. Figure 1 illustrates the course of double fixation. In group B, the clavicle was fixed with beach-chair position and open approach with the same superior plating technique. Radiographs were taken immediately after treatment, at 4 weeks, 8 weeks, 3 months and then subsequently every 6 months for 2 years. Union of both fracture sites and the GPA after fracture consolidation were assessed by plain radiographs. The mean GPA measured by previous three same physicians was taken for analysis. The Disabilities of the Arm, Shoulder and Hand (DASH) score [14] and Constant scores [15] were used to evaluate the functional recovery of patients. The healing time for clavicle and scapular neck were also recorded and compared between the three groups.

### Statistical analysis

Patients’ GPAs were measured by three physicians, and the mean GPA was used for data analysis. The measurement of reliability GPA was examined by intra-class correlation coefficient (ICC). Nonparametric methods were used to compare the differences in continuous variables and Fisher’s exact test was for categorical variables among the three treatment groups. Bonferroni’s *post hoc* method was used to perform pair wise comparisons of the groups. In addition, linear regression models were fitted for DASH score and Constant score with the post-operative GPA; the areas under the receiver operating characteristic (ROC) curve were also performed to evaluate that the post-operative GPA predicts DASH and Constant scores. We implemented a generalized ROC curve proposed by Obuchowski et al. [16] to estimate the accuracy of a test when the gold standard is measured on a continuous scale. All data processing and statistical analyses were performed with SAS software version 9.3 (SAS Institute, Inc., Cary, NC, USA), except the ROC analysis which was carried out by R “nonbinROC” package [17]. Statistical significance was defined as *p* < 0.05.

### IRB approval

The study was approved by the local IRB/Research Ethics Committee: CMUH102-REC2-062.

## Results

### Patient characteristics

Among the 39 patients, 26 (67%) were injured in traffic accidents, 7 (18%) falling from a height, and 6 (15%) falling from a bicycle. Thirty-two patients had multiple injuries. Associated injuries in these multiple injury patients included head injury (*n* = 11), fracture of one or more ribs (*n* = 24), hemo-pneumothorax (*n* = 8), abdominal injury (*n* = 4), and fracture of one or more extremities (*n* = 22).

Twenty-two fractures were on the right side and 17 were on the left. The mean interval between time of injury and shoulder operation was 5 (2 to 8) days. There were no post-operative infections or neurovascular injuries. Background information for all subjects is presented in Table 1.

**Table 1:** **Selected characteristics of the patients**

**Variables**	**Treatment group**						
	**A (** ***n*** **= 13)**		**B (** ***n*** **= 13)**		**C (** ***n*** **= 13)**		
	***n***	**%**	***n***	**%**	***n***	**%**	***p*** ** value**
Sex							
Females	7	53.85	3	23.08	6	46.15	0.3552
Males	6	46.15	10	76.92	7	53.85	
Glenopolar angle (GPA) after consolidation							
Normal (30°–45°)	9	69.23	7	53.85	4	30.77	0.1787
Abnormal (otherwise)	4	30.77	6	46.15	9	69.23	
	Mean	SD	Mean	SD	Mean	SD	*p* value
Age	38.77	17.71	43.23	10.14	44.92	18.35	0.5970
Injury Severity Score (ISS)	11.38	7.95	13	10.17	23.92	18.09	0.0354
Glenopolar angle (GPA) at injury	15.08	8.06	25.92	9.88	24.92	11.15	0.0133
Glenopolar angle (GPA) after consolidation	35.54	7.10	28.23	8.88	25.23	10.17	0.0152
Disabilities of the arm, shoulder and hand score	1.22	1.93	23.24	25.32	20.81	19.39	0.0078
Constant-Murley shoulder outcome score	76.57	5.46	65.52	11.04	62.35	11.31	0.0016
	**Adjusted mean** ^**a**^	**SE**	**Adjusted mean** ^**a**^	**SE**	**Adjusted mean** ^**a**^	**SE**	***p*** ** value**
Disabilities of the arm, shoulder and hand score	2.49	5.60	26.52	4.58	16.28	5.14	0.0092
Constant-Murley shoulder outcome score	75.22	3.15	64.51	2.58	64.86	2.90	0.0479

^a^Adjustment of age, gender, ISS, and GPA at injury.

*SD* standard deviation, *SE* standard error.

The union time of the scapular neck was shorter in group A (median: 10 weeks), compared to group B (median: 12 weeks, *p* = 0.01) and group C (median: 12 weeks, *p* = 0.036). However, the difference in the union time of the clavicle among the three groups was not statistically significant (*p* = 0.745).

All patients were available for follow-up for 2 years. After bone union, the difference in GPA was still significant among the three groups (median = 36°, 28°, and 25° for groups A, B, and C, respectively; *p* = 0.015). DASH score was significantly better (a lower value) in group A than that in groups B and C (*p* = 0.008); while DASH score was not significantly different between groups B and C (*p* = 0.738). The similar relation was observed in Constant score among the three treatment groups, although a higher value of a Constant score means a better result.

After the adjustment of age, sex, ISS, and GPA at injury, GPA, DASH, and Constant scores after fracture consolidation still differed significantly among the three treatment groups (*p* = 0.001, 0.0092, and 0.0479, respectively) (Table 1). Group A (fixation of both the clavicle and the scapular neck) demonstrated a better outcome in GPA and DASH and Constant scores without and with the adjustment of other baseline variables.

**Figure 2:**
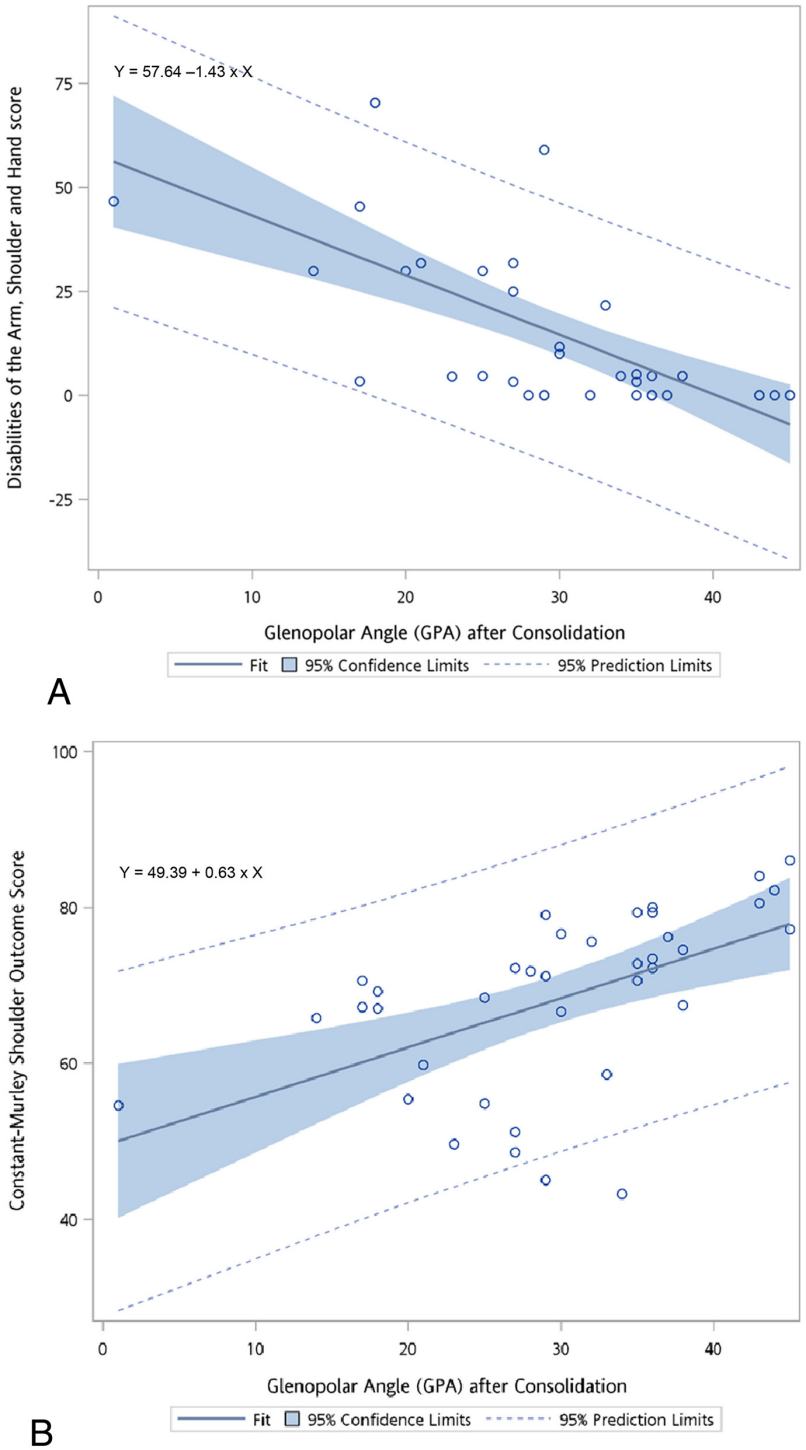
**The simple linear regression model of (A) the Disabilities of the Arm, Shoulder and Hand (DASH) score and (B) the Constant-Murley Shoulder Outcome (Constant) score with the post-operative glenopolar angle (GPA).**

GPA and DASH score were highly negatively correlated (Spearman rank correlation coefficient = −0.70, *p* < 0.001). In contrast, GPA and Constant score were highly positively correlated (Spearman rank correlation coefficient = 0.73, *p* < 0.001). The simple linear regression model demonstrated that 1° increase in the post-operative GPA would decrease 1.43 points of DASH score but increase 0.63 point of Constant score (Figure 2).

The ROC analysis for a continuous gold standard showed that of two random DASH scores, there was a 75.1% chance (standard error 3%) that the smaller DASH score would have a larger GPA than the larger DASH score. Similarly, there was a 73.4% chance (standard error 4%) that the larger Constant score would have a larger GPA than the smaller Constant score.

### Measurement reliability

The GPAs were measured by three different physicians. The mean GPA for 39 patients was 22.05° (SD = 10.69), 21.95° (SD = 10.90), and 21.97° (SD = 10.71) at injury and 28.51° (SD = 11.00), 29.31° (SD = 10.76), and 28.97° (SD = 10.75) after fracture consolidation. The ICC was 0.99 for GPA at injury and after fracture consolidation.

## Discussion

Malunion or nonunion of the scapular neck are associated with a poor functional outcome with complications of weak abduction and subacromial bursitis [18]. These symptoms are attributed to loss of a normal rotator cuff lever arm. Chadwick et al. [19] found that scapular neck malunion changed the working lengths of muscles crossing the shoulder, especially the rotator cuff complex. The current study revealed fixation of both the clavicle and the scapular neck can achieve better correction of GPA and clinical outcomes of DASH and Constant scores than either fixation of the clavicle alone or conservative treatment. The restoration of GPA also highly positively correlated with better DASH and Constant scores from the Spearman rank correlation and ROC analysis. Numerous studies have looked at the results of both operative and conservative management of floating-shoulder injuries, and there has been evidence in favor of both treatments [1-6,8,9,20-22]. That is, diverging opinions are reported regarding the choice of treatment of floating-shoulder injuries. Guidelines for this injury are controversial because of small numbers of patients and because there has been no uniformity in the measures used to assess outcomes of treatment. The GPA, 30° to 45° is considered normal, quantifies the obliquity of the glenoid articular surface in relation to the scapular body and can be used to assess rotational malalignment of the glenoid neck [23].

Our results of correlation between GPA and clinical outcomes are in line with the related literature. Bozkurt et al. [24] found a highly positive correlation between functional outcome and GPA: Constant score decreased as GPA decreased. They also concluded that when used to determine the degree of instability, decreased GPA has been proven to be more reliable than the fracture type itself. Kim [25] found that GPA correlated with clinical outcome of floating-shoulder injuries. Romeo et al. [12] suggested that a less favorable outcome is expected if there is persistence of rotational malalignment of the glenoid, especially when GPA is less than 20°.

In the current study, the post-treatment GPA of clavicular fixation alone revealed no total realignment of the glenoid in relation to the scapular body. Correction of median GPA from 18° pre-operatively to 36° after fracture consolidation could be achieved only in both fixation scapula and clavicle. In only the clavicle fixation group, indirect reduction of scapular neck fracture after anatomic reduction of the clavicle was not seen, noting a median GPA of 21° pre-operatively to 30° after fracture consolidation. Restoration of the anatomical shape of the clavicle may automatically reduce the displacement of a scapular neck fracture only in a minimally displaced scapular fracture, as shown in the series of Hashiguchi and Ito [21], in cases with minimal displacement of scapular neck fracture. If clavicular internal fixation alone is performed in such problematic injuries, the scapular neck fracture will not be stable and functional disorders of the rotator cuff may occur as a result of malunion or nonunion of the scapular neck [9]. Izadpanah et al. [26] strongly recommended that primary fixation of the scapula be performed in patients with a post-injury GPA of less than 30° which was also supported by Kim’s study [25].

There are a number of limitations to our study that are worth highlighting. First, there were limited numbers of patients in each group because of the relative rarity of this injury. However, the importance of this study is that it is a large series in the literature that compares the influences of three different treatments in patients with floating-shoulder injuries on GPA and DASH and Constant scores. Another limitation was higher ISS in the conservative treatment group and lower GPA in the double fixation group even with the stratified randomized nature of the study. We adjusted the factors of age, gender, ISS, and GPA at injury to analyze DASH and Constant scores. Besides, DASH and Constant scores specifically evaluate upper limb function, and our exclusion criteria for patient selection were stringent, implying that DASH and Constant scores were affected mainly by the floating shoulder itself.

Our study indicates that fixation of both the clavicle and the scapular neck may correct GPA and improve functional outcomes of floating-shoulder injuries better than either fixation of the clavicle alone or conservative treatment. GPA after fracture consolidation is a useful prognostic indicator of a satisfactory clinical outcome as defined by either DASH score or Constant score.

## Abbreviations

GPA: Glenopolar angle
DASH: Disabilities of the Arm, Shoulder and Hand
ISS: Injury severity score
SSSC: Superior shoulder suspensory complex
AP: Anteroposterior
ROC: Receiver operating characteristic
CI: Confidence interval
SEN: Sensitivity
SPE: Specificity
SD: Standard deviation
